# Marine Observation Beacon Clustering and Recycling Technology Based on Wireless Sensor Networks

**DOI:** 10.3390/s19173726

**Published:** 2019-08-28

**Authors:** Zhenguo Zhang, Shengbo Qi, Shouzhe Li

**Affiliations:** 1 College of Engineering, Ocean University of China, Qingdao 266100, China; 2 College of Letters and Science, University of Wisconsin-Madison, Madison, WI 53711, USA

**Keywords:** wireless sensor networks, k-means algorithm, network energy, observation beacon, fuzzy logic system

## Abstract

Monitoring of marine polluted areas is an emergency task, where efficiency and low-power consumption are challenging for the recovery of marine monitoring equipment. Wireless sensor networks (WSNs) offer the potential for low-energy recovery of marine observation beacons. Reducing and balancing network energy consumption are major problems for this solution. This paper presents an energy-saving clustering algorithm for wireless sensor networks based on k-means algorithm and fuzzy logic system (KFNS). The algorithm is divided into three phases according to the different demands of each recovery phase. In the monitoring phase, a distributed method is used to select boundary nodes to reduce network energy consumption. The cluster routing phase solves the extreme imbalance of energy of nodes for clustering. In the recovery phase, the inter-node weights are obtained based on the fuzzy membership function. The Dijkstra algorithm is used to obtain the minimum weight path from the node to the base station, and the optimal recovery order of the nodes is obtained by using depth-first search (DFS). We compare the proposed algorithm with existing representative methods. Experimental results show that the algorithm has a longer life cycle and a more efficient recovery strategy.

## 1. Introduction

**Figure 1:**
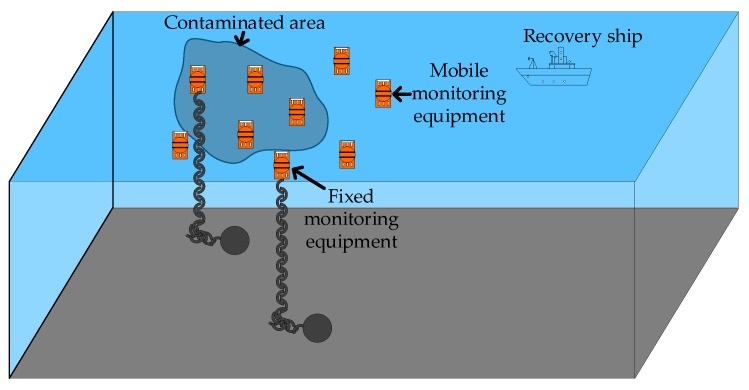
Schematic diagram of marine environmental buoy monitoring.

The utilization of marine resources is increasingly becoming important and active to solve the issue of resource shortage. Correspondingly, marine pollution is becoming more serious with the exploitation of marine resources [1,2]. Wireless sensor networks (WSNs) play an important role in oceanic scenarios such as environmental monitoring and ocean exploration [3,4,5,6,7]. The categories of ocean monitoring include marine buoy monitoring, vessels, satellites, and aerial-based monitoring [8,9,10]. Compared with other detection methods, mobile and fixed-point ocean observation buoy monitoring has advantages in accuracy and cost performance [11]. After an ocean observation beacon completes the data collection task, it needs to be recycled, as shown in Figure 1. However, in the process of monitoring the marine environment, due to the influence of natural hazards such as waves and tides on the monitoring equipment, there are still many problems in completing the recovery of large-scale monitoring equipment [12,13]. So far, clustering is the most effective way to improve target detection efficiency [14]. Meanwhile, due to the instability of the marine environment, the real-time location of the monitoring equipment directly affects the communication efficiency and recovery efficiency of the equipment [15]. Therefore, the rapid acquisition of node information is essential for the recovery of the target.

WSNs have different requirements for routing algorithms, according to different application scenarios. The main purpose is to extend the network life cycle and balance the energy consumption of network nodes [16]. WSNs are composed of a number of sensor nodes (SNs), which are generally powered by lithium batteries and communicate wirelessly to form a sensor communication network. Because the nodes are powered by batteries, it is not realistic to replace the dead nodes with batteries in a wide range of application scenarios. Therefore, the energy problem is a key factor limiting the reliability of WSNs [17,18,19,20]. The low-power design of the node is an effective way to solve the energy problem. However, low-power design limits the computational and storage capabilities of the node [21,22].

In these protocols, SNs are divided into ordinary nodes and cluster heads (*CH*s). An ordinary node does not directly communicate with the base station (BS), but sends information to the *CH*s, k whereas the *CH*s send the processed data to the BS [23,24,25]. Therefore, the *CH* forwarding not only reduces the energy consumption of the node’s ultra-long-distance transmission, but also the data transmission amount through data fusion. The clustering algorithm is divided into centralized and distributed according to the way the *CH* is generated [26]. The centralized algorithm selects the *CH* based on the global information of the network, and the distributed algorithm generally adopts the method of “self-recommended” or partial competition to generate the *CH*. In the cluster-based approach, the rationality of clustering and *CH* allocation can ensure the balance of network energy consumption, network scalability, and manageability.

Many researchers proposed methods for solving the energy problems of WSNs. The low-energy adaptive clustering hierarchy (LEACH) algorithm is the most classical clustering algorithm [27]. The node rotation mechanism is used to select the *CH*. Each node has the opportunity to be elected as the *CH*. The *CH* selection mechanism of the algorithm cannot guarantee the quantity and quality of the *CH* and does not consider the node energy problem. The network is prone to the “energy hole” phenomenon. The power-efficient gathering in sensor information systems (PEGASIS) algorithm is based on the idea of the LEACH algorithm, which constructs a chain through greedy algorithms for all nodes in the network [28]. The algorithm requires each node to know the location of other nodes, which increases the storage difficulty of nodes. The long chain length increases the communication energy consumption of the node, and *CH* failure leads to network routing failure. The LEACH-C (LEACH centralized) algorithm effectively compensates for the shortcomings of LEACH algorithm by selecting *CH*s through the BS [29]. However, the flexibility of the algorithm is limited by the availability of the global information. The distributed energy-efficient clustering (DEEC) algorithm adds the initial energy and residual energy of the node based on the LEACH-C algorithm. The DEEC algorithm not only balances network energy but also extends the network life cycle [30]. The algorithm requires uniform network energy consumption, which limits the practicability of the algorithm. The LEACH-MEEC (LEACH mobile, energy-efficient, and connected) algorithm selects the *CH* by the connectivity between adjacent nodes and the residual energy of the nodes. The LEACH-MEEC algorithm uses four mobile models to verify the rationality of the algorithm [31]. The *CH* of each round is selected according to the state of adjacent nodes, which lacks global features. The energy-efficient unequal clustering (EEUC) algorithm adopts a networking method of non-uniform clustering and inter-cluster multi-hop routing [32]. The distance between the *CH* and the BS is taken into consideration when selecting the *CH*. In order to balance the network energy, the distance between the relay node and the BS and the energy of the relay node need to be considered when selecting the relay node. The *CH* selection mechanism of the algorithm does not add the node energy factor, which has some defects. The ant colony optimization-based uneven clustering (ACOUC) algorithm is an improved version of EEUC algorithm, which adds a directional diffusion ant colony optimization algorithm to the EEUC algorithm [33]. The algorithm can yield more surviving nodes in a longer period of time. The ACOUC algorithm has extremely complex message complexity. When the network size is large, there is a heavy network burden. The coverage and energy-aware clustering algorithm (CECA) guarantees the rationality of the *CH* and cluster compactness based on parameters such as node degrees, distance between nodes, and energy [34]. However, the rationality of the cluster size cannot be guaranteed.

Heuristic algorithms and clustering algorithms provide a better solution for reducing the energy consumption of WSNs. Reference [35] used the k-means algorithm to optimize the LEACH and HEED (hybrid energy-efficient distributed clustering) algorithms to increase clustering compactness of the network based on Euclidean distance. Simulation results showed that the scheme improves both network lifetime and energy efficiency. The two-tier distributed fuzzy logic-based protocol (TTDFP) is a two-layer distributed fuzzy logic algorithm. The first layer of the algorithm selects *CH*s through the energy competition of the provisional leaders. The second layer uses the three connectivity parameters (node connectivity, distance to the BS, and remaining node energy) to find the optimal path from the channel to the receiver. The clustering method of the algorithm is a fuzzy distributed non-uniform clustering method. There is no BS involvement in the *CH* election process [36]. The improved energy-efficient cluster head selection (IEECHS) algorithm selects two *CH*s in a single cluster and proposes corresponding data fusion techniques according to the characteristics of the dual *CH*s. Experiments showed that this method has good performance in terms of network lifetime and energy consumption [37]. The energy-balance routing protocol (EBRP) uses the k-means++ algorithm to divide the network into clusters and uses fuzzy logic systems (FLS) to optimize *CH* selection. The algorithm uses the genetic algorithm (GA) to obtain fuzzy rules [38]. The simulation results showed that the EBRP algorithm has a longer life cycle than the current routing protocol network. The FL-EEC/D (fuzzy logic-based energy-efficient clustering for WSN based on minimum separation distance) algorithm uses a k-means-based fuzzy logic *CH* selection model using descriptors such as residual energy, position suitability, density, compression, and distance between nodes and the BS [26]. The algorithm uses the Gini index to measure energy efficiency. In Reference [39], an adaptive neuro fuzzy inference system (ANFIS) and an artificial bee colony (ABC) algorithm were proposed to solve the problem of dangerous goods path planning. The study sought the optimal path by considering the combination of seven factors (operating costs, emergency response, risk associated with the environment, etc.). The Dijkstra risk (D-R) model is a new method based on the combination of multi-standard risk analysis and the traditional Dijkstra algorithm [40]. The model uses a variety of potential factors and normalizes the indicators to optimize path selection. The energy saving-oriented least-hop routing algorithm (ESLHA) is based on improvements of Dijkstra’s (ESRAD) energy-efficient routing algorithm [41]. The algorithm introduces node processing information energy consumption and inter-node information transmission energy consumption to evaluate node indicators. Then, the minimum energy consumption path is obtained by the Dijkstra algorithm. Reference [42] used the weighted sum method to calculate node weights, and the node closest to the standard weight of a particular cluster was elected as *CH*. The Dijkstra algorithm was used to obtain the optimal path for information transfer. However, GA is computationally intensive and fuzzy rules apply simple if–then rules to distribute computing whether it is machine learning, other intelligent algorithms, or natural-based heuristic evolutionary algorithms [43], which require nodes and BSs with high computing performance and high memory capacity. In particular, when the number of networks is large, the system delay increases [26]. Therefore, it is necessary to limit the network scale or application range for increasing system real-time performance.

The communication efficiency and recovery efficiency of the nodes are challenges as discussed in the previous discussions. To target the reduction of node energy consumption on communication and to increase node recovery efficiency, we propose the k-means algorithm and fuzzy logic system (KFNS), a data gathering algorithm for the recycling of marine beacons. Compared with published methods, the algorithm takes the advantages of centralized and distributed algorithms. It not only increases the real-time performance of the system but also reduces unnecessary data communication to improve network lifetime. Another highlight of this work is that the algorithm proposed in this paper has lower hardware requirements for nodes. We take into account the low power consumption factor and assign the nodes with simplified tasks. We also introduce the FLS and Dijkstra algorithms for optimal path selection with certain adaptability. Path selection weights are determined by a variety of influencing factors.

The KFNS algorithm is divided into three stages: Monitoring phase, cluster routing phase, and recovery phase. The KFNS algorithm discards network construction in the monitoring phase. The boundary nodes are selected by Euclid distance due to the limited computing power of the nodes to maximize network energy. In the cluster routing phase, the initial clustering center and the number of clusters are selected according to the location of the boundary nodes and the real-time requirements of the system to optimize the clustering effect and extend the network life cycle. In the recovery phase, a single cluster recovery strategy is adopted; the FIS is used to optimize the Dijkstra algorithm; the DFS is used to obtain the optimal recovery order of the nodes and accelerate the recovery efficiency of monitoring equipment. The application background of the marine observation beacon recycling has its unique problems compared to other WSNs application scenarios. The KFNS algorithm combines application scenarios and phased target requirements to better suit actual requirements. The method optimizes network energy consumption and improves network performance through these phase divisions. The main contributions of this paper are given below.

♦ We divide the recycling process of the marine observation beacon into three phases. The algorithm is designed to meet the demands of different phases.
♦ A novel scheme is proposed where the FLS is used to comprehensively consider the influence of various environmental factors on the path weight, breaking through the limitations of traditional description methods.
♦ We propose an effective solution by using centralized and distributed algorithms. After the BS completes the clustering, the *CH* replacement is completed by the nodes in the cluster. Nodes reduce unnecessary communication energy consumption, which extends the network life cycle.

The rest of this paper is organized as follows: Section 2 introduces the network model, including the node model, the energy model, and the node movement model. Section 3 introduces the proposed recovery algorithm for large-scale ocean observation beacons based on WSNs. Simulations and experiments are shown in Section 4, and Section 5 concludes this paper.

## 2. Network Model

In this section, the node model, energy model, and node movement model are presented in detail [44].

### 2.1. Node Model

(1) This paper assumes that nodes are distributed over a continuous two-dimensional plane. This plane has no isolated points (beyond the communication range of all other nodes).
(2) The node uses the LoRa (Long Range Radio) module to communicate, and the nodes in different clusters can be simultaneously communicated by changing the LoRa frequency band.
(3) Node information: *n* nodes are randomly and independently distributed in a circular area. The size of the area is $\pi R^{2}$, where *R* is the radius. Node information is represented by $S=\{S_{1},S_{2}\cdots S_{n}\}$, and the initial energy of the node is $E_{i}=E_{0}\times (0.9+rand*(0.1))$, where $E_{0}=5$. Due to the difference between the beacon battery and the beacon start-up time, the initial power of each node is different.
(4) The node controls the node communication range by controlling the transmission power.
(5) All nodes are positioned and calibrated periodically by a global positioning system (GPS).
(6) Each node has a unique identifier (ID) number and has small computing and storage capacity.
(7) It is assumed that the *CH* receives *k* bits of data from each node and can be compressed into k bits of data.

### 2.2. Energy Model

All nodes satisfy the free communication model [45]. The node communication energy consumption includes sending transmission consumption and receiving energy consumption. The transmission energy consumption includes the energy consumption of the RF (Radio Frequency) module and the signal amplification; the receiving energy consumption includes the energy consumption of the receiving module. When the communication distance is less than the distance threshold, $d_{Threshold}$, the free space propagation model is adopted, and the path attenuation index is 2. When the communication distance is greater than the threshold, $d_{Threshold}$, the two-ray propagation model is adopted, and the path attenuation index is 4.

**Figure 2:**
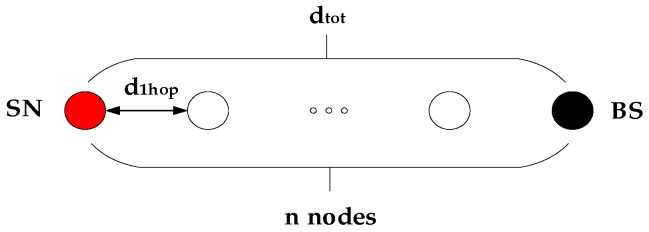
Node communication model.

The node transmits *k* bits of data through multi-hop routing between clusters, as shown in Figure 2 and its energy consumption is as follows [46]:

(1) $$P\approx k\times ceil(\frac{d_{\mathrm{tot}}}{d_{1hop}})\times (2E_{elec}+E_{\mathrm{cpu}}+E_{\mathrm{amp}}\times d_{1hop}^{\gamma }),$$

where P is the energy consumption for sending *k* bits of data, $d_{tot}$ is the distance from the sending point to the target node, $E_{elec}\left(nj/bit\right)$ is the RF energy consumption coefficient, $E_{amp}\left(nj/bit/m^{2}\right)$ is the amplifier energy factor, $E_{cpu}$ is processor power consumption, $d_{1hop}$ is the distance between neighbor nodes and γ is the signal attenuation index.

The optimal single-hop distance obtained from Equation (1) is as follows:

(2) $$d_{1hop}=\sqrt[{\gamma }]{\frac{k\times d_{tot}\times (2E_{elec}+E_{cpu})}{E_{amp}\times (\gamma -1)}}.$$

To ensure that the signal-to-noise ratio (SNR) is within a reasonable range, the energy consumption model of the node sending data is calculated as follows:

(3) $$E_{Tx}(k,d)=\{\begin{array}{c} E_{elec}\times k+E_{fs}\times k\times d^{2},d\leq d_{Threshold}\\ E_{elec}\times k+E_{mp}\times k\times d^{4},d>d_{Threshold} \end{array}.$$

The energy consumption model of the node receiving data is determined as follows:

(4) $$E_{Rx}=E_{elec}\times k,$$

where $E_{Tx}$ is the transmission energy consumption, d is the transmission distance, $E_{fs}\left(nj/bit/m^{2}\right)$, $E_{mp}\left(nj/bit/m^{4}\right)$ is the power dissipation factor of the amplifier under different communication models, $d_{Threshold}$ is the distance threshold, and $E_{Rx}$ is the receiving energy consumption.

### 2.3. Node Movement Model

In the marine environment, the simulation results based on the mobile model are reliable [47]. The entity movement model includes the following aspects:

(1) Random walk;
(2) Random waypoint mobile model;
(3) Random direction model;
(4) Gauss Markov model.

In the field environment, the velocity and direction before and after the joint motion interact with each other. The Gauss Markov model can better describe the motional behavior of the node. The Gauss Markov model assigns an initial velocity and initial direction to each node. After a fixed interval, the node updates its current speed, direction, and location information as follows:

(5) $$s_{n}=\alpha s_{n-1}+(1-\alpha )\bar{s}+\sqrt{\left(1-\alpha^{2}\right)}\gamma_{m},$$

(6) $$s_{n}=\alpha s_{n-1}+(1-\alpha )\bar{s}+\sqrt{\left(1-\alpha^{2}\right)}\gamma_{m},$$

where $s_{n}$ and $d_{n}$ are the speed and direction of the node at time *n*, $\bar{s}$ and $\bar{d}$ are the average value of the speed and direction, $\gamma_{m}$ and $\varphi_{m}$ are random variables subject to Gaussian distribution, and α is a randomness variable generally taken as 0≤α≤1.

(7) $$x_{n}=x_{n-1}+s_{n-1}\cos d_{n-1},$$

(8) $$y_{n}=y_{n-1}+s_{n-1}\sin d_{n-1},$$

where $\left(x_{n},y_{n}\right)$ are the coordinates of the node at time *n*, and $\left(x_{n-1},y_{n-1}\right)$ are the coordinates of the mobile node at time *n*−1.

## 3. Proposed KFNS Algorithm

In the recovery process of the marine observation beacon, it can be further divided into three stages according to different requirements including: the monitoring phase, the cluster routing phase and the recovery phase. The corresponding algorithm is designed over the characteristics of each stage to improve the applicability of the algorithm in the recycling process.

We define $D_{BS}$ as the distance between the BS and the node network, $D_{\max}$ as the distance between the BS and the node network when it is greater than the communication distance, and $D_{\min}$ as the range in which the BS enters to recover the node. 

**Figure 3:**
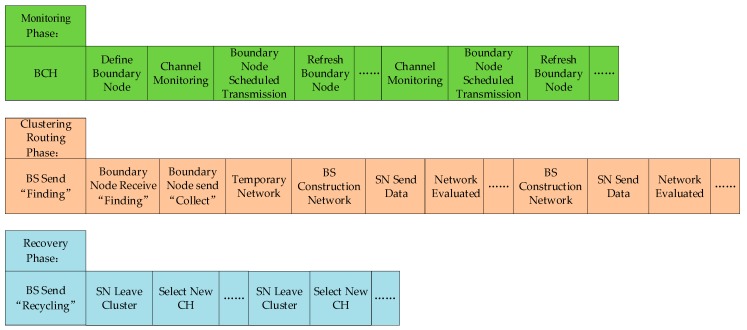
K-means algorithm and fuzzy logic system (KFNS) workflow at each stage.

**Figure 4:**
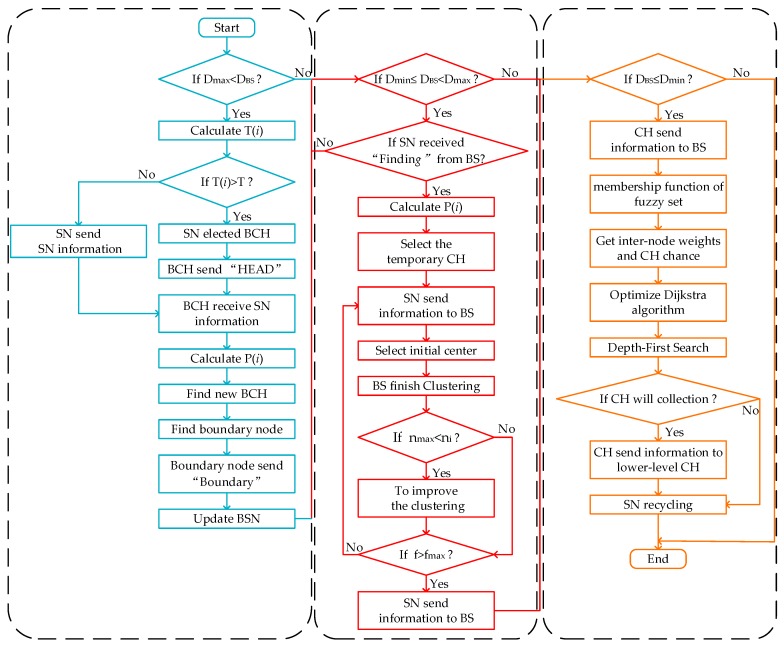
KFNS algorithm flow chart.

When the BS does not reach the node network boundary, the node network is in the monitoring phase ($D_{\max}<D_{BS}$). The main purpose of this phase is to monitor whether the BS reaches the periphery of the node networks and the internal nodes of the network are in sleep mode. Therefore, the network can maximize its energy. When the BS moves to the node network boundary, the node is in the cluster routing phase ($D_{\min}\leq D_{BS}<D_{\max}$). This phase is to complete the node networking operation. When the BS can summarize the beacon location information. The node network information can provide data support for the direction of movement of the BS. When the BS moves to the node recovery range, the node network is in the recovery phase ($D_{BS}\leq D_{\min}$). The main goal of this phase is the dynamic update of the CH and the order in which the nodes are recycled. Using fuzzy rules can break through the limitations of traditional assessment methods. Figure 3 and Figure 4 are the work flow of each stage and the flow chart of KFNS algorithm respectively. 

### 3.1. Monitoring Phase

When $D_{\max}<D_{BS}$, we set the initial mode of the node to monitoring mode. The node firstly compares its own residual energy and initial energy conditions with the set threshold, as well as the energy consumption ratio over time. When T(i) is greater than the set threshold *T*, the node acts as a boundary cluster head (BCH). Based on the relationship between the ID number and the time *t*, the BCH sends the contention message “HEAD” to the neighbor node with the optimal communication distance as follows:

(9) $$t=(T_{rec}+T_{send}+T_{cpu}+T_{delay})\times ID,$$

where t is the node information interaction and processing time, $T_{rec}$ is the time when the node receives data, $T_{send}$ is the time when the node sends data, $T_{cpu}$ is the data processing time, and $T_{delay}$ is the anti-collision delay.

When the BCH receives the message from other BCHs, the receiver gives up the *CH* identity. The broadcast time interval of the same BCH is then calculated as follows:

(10) $$T_{n+1}-T_{n}>n\times t,$$

where *n* is the number of nodes to guarantee that only one BCH works at a time. When the same BCH broadcasts more than a certain number of times, but there is no neighbor node response, then the BCH considers leaving the network and enters sleep mode until the periodic wake-up. The probability that a node is elected as a BCH is as follows:

(11) $$T(i)=\alpha E_{i\_current}+(1-\alpha )\frac{E_{i\_\mathrm{sta}rt}-E_{i\_current}}{t_{i\_now}-t_{i\_start}},$$

where T(i) is the probability that node i is elected as the BCH, $E_{n\_current}$ is the current node energy, $E_{n\_\mathrm{start}}$ is the initial energy of the node, $t_{now}$ is the current time of the node, $t_{\mathrm{start}}$ is the node boot time, and α is the proportion of energy and time.

After the surrounding node receives the BCH information, if the current neighbor node is configured to be a BCH, the neighbor node abandons the BCH identity. All neighbor nodes send their own location information and node energy information to the BCH in TDMA (Time Division Multiple Access) mode with their ID number after the BCH receives the information of the neighbor node. According to the position of the receiver, the optimal communication distance is the radius, and the fan-shaped area with an angle of 90° is divided into four regions. The average energy of the nodes in the current region is firstly calculated. Then, according to the energy and distance of the neighbor nodes in the current domain, the probability that all neighbor nodes in the region become temporary boundary nodes is obtained. The neighbor node with the highest probability becomes the temporary boundary node of the domain, and the probability is calculated as follows:

(12) $$P(i)=\alpha \frac{E_{i}}{E_{ave}}\times (1-\alpha )\frac{d_{iBCH}}{d_{ave}},$$

where P(i) is the probability that the node is elected as a temporary boundary node, $E_{i}$ is the current remaining energy of the node, $E_{ave}$ is the average energy of all nodes in the four regions of the BCH, $d_{iBCH}$ is the distance between the boundary node and the BCH, $d_{\mathrm{ave}}$ is the average distance from all nodes to the BCH, and α is the specific gravity relationship of each parameter.

According to Equation (12), temporary boundary nodes of four regions of BCH can be obtained. The BCH broadcasts regional network energy and temporary boundary node information to nodes in the area. The BCH abandons the *CH* identity and enters sleep mode (timed wake up to receive messages). The intra-area node receives the information of the BCH and then classifies it as a normal node or a temporary boundary node. The normal node enters sleep mode, and the temporary boundary node becomes the new BCH. The latest generated BCH continues to broadcast the “HEAD” message and receives node information for the other three regions. The process is repeated in turn until the outermost corner of the network. When the BCH does not have a suitable temporary boundary node selection in a certain area, the BCH acts as a boundary node.

Because the nodes move relative to each other, the boundary nodes need to broadcast “boundary” messages periodically. In order to improve communication success rate and reduce signal collision, firstly, the radio signal of the air is monitored by the channel monitoring function of the LoRa module. When there is a radio signal in the air, it continues to monitor after a random delay. If there is no signal communication in the air after the delay, the “boundary” message is broadcasted. Secondly, the default zone node information is received if new node information appears. A new boundary node is generated based on the distance and energy of the neighbor nodes. If no new boundary node is generated, the boundary node enters the receiving mode, and other ordinary nodes enter the sleep mode. When the energy of the boundary node is lower than 15%, the boundary node sends the final sleep message, and the boundary node identity is abandoned to enter the sleep mode (waking up regularly to receive the message). After receiving the final sleep message of the boundary node, the neighbor node re-selects the boundary node.

### 3.2. Cluster Routing Phase

#### 3.2.1. Temporary Network Routing

The BS periodically broadcasts the “finding” message. After the packet is received by the boundary node, it sends its own location and energy information to the BS $\left(D_{\min}\leq D_{BS}<D_{\max}\right)$. The boundary node informs the BS that it reached the edge of the network and the BS continues to move in the direction of the network target. After the boundary node sends its own location energy information, it broadcasts a “collect” message to collect network node location and energy information for temporary networking. Due to the low-power design, the microprocessor has limited computing capability. Therefore, the temporary networking algorithm cannot be too complex. After receiving the collection command, the neighbor node sends its own position and energy information to the boundary node in TDMA with its own ID. The boundary node selects the temporary *CH* of each domain according to the average energy of the neighbor node and the distance from the boundary node. The distance factor from the node to the temporary *CH* and the energy consumption rate of the node are introduced in the election of a temporary *CH*. The node that makes the most energy far away from the BS is elected as the temporary *CH*, and the probability of being elected as the temporary *CH* is calculated as follows:

(13) P(n)=αEn_currentEn_start+βdiBS∑i∈CHRed(ni,CHRe)N+γEn_start−En_currenttnow−tstart,

(14) α+β+γ=1,

where P(n) is the probability that a node is elected as a temporary CH, $E_{n\_current}$ is the current remaining energy of the node, $E_{n\_start}$ is the initial energy of the node, $t_{now}$ is the current node time, $t_{\mathrm{start}}$ is the node startup time, $d_{iBS}$ is the distance from the node to the temporary CH, $d\left(n_{i},CH_{RE}\right)$ is the distance from the node to the temporary CH, and α,β,γ are the proportion of each part.

After the boundary node selects the temporary *CH*, the temporary *CH* broadcasts the “HEAD” message. Neighbor nodes match “HEAD” messages to join temporary clusters according to their ID. The next suitable temporary *CH* is selected by Equation (13) according to the neighbor node information until all nodes form a temporary network. The temporary network sends information to the BS through a multi-hop route. Because the BS has no energy limitation and has certain computing power, the BS clusters the nodes by summarizing the node information of the entire network.

#### 3.2.2. Enhanced K-Means Algorithm

The traditional k-means algorithm is a simple iterative clustering algorithm, which classifies all points into *k*-class by selecting *k* initial centers. The algorithm selects the optimal clustering center by iteration and compares the effect of each iteration with that of the previous iteration. Until the difference between the two iterations is within the set threshold, the iteration is completed [48,49]. The traditional k-means algorithm needs to specify the number of clusters *k*, which requires a certain prior condition. an improper value of *k* not only affects the clustering effect but also causes uneven energy consumption of nodes. The initial clustering center is randomly selected to increase the number of clustering iterations and affect the clustering effect.

Combined with the application background of the marine observation beacon, the deficiencies of the traditional k-means algorithm are improved. The improvement ideas are as follows: (1) the number of clusters *k* is determined according to the real-time requirements and empirical values of the system. The clustered optimal solution is found by comparing the clustering compactness functions after different *k* value iterations. (2) The initial cluster center is determined according to the location relationship of boundary nodes. The intra-cluster clustering is implemented by adding the intra-cluster evaluation effect function, and the real-time processing of the information in the cluster is increased. Therefore, it can maximize the rationality of clustering results, prolong network lifetime, and optimize the energy consumption of boundary nodes and *CH*s. The improvement process is described by Algorithm 1.

**Algorithm 1** Improved *k*-means algorithm**Input**: E={P,Q}, $P=\left\{p_{1},p_{2},\cdots ,p_{i}\right\}$, $Q=\left\{q_{1},q_{2},\cdots ,q_{j}\right\}$//set of i ordinary sensor nodes and *j* boundary sensor nodes.**Output**: A set of *k* clusters $C=\left\{C_{1},C_{2}\cdots C_{k}\right\}$
1: **for**
i
←
$k_{\min}$ to $\sqrt{n}$ do2: $C_{i}\leftarrow \emptyset$
3:  choose centroid $r_{i}$ among E belong to $C_{i}$
4:  **for** each set $E_{j}\in E$
**do**5:    assign $E_{j}$ to the cluster $C_{i}$ with nearest $r_{i}$ i.e. $\left(dis_{j,i}\left(E_{j},r_{i}\right)\leq dis_{j,i}\left(E_{j},r_{i*}\right);i\in \left\{k_{\min},\cdots ,\sqrt{n}\right\}\right)$
6:  **end for**7:  **repeat**8:  **for** all $i\in \left(k_{\min},\cdots ,k_{opt}\right)$ and cluster $C_{i}$
**do**9:   the centroid $r_{i}$ to be the center of all nodes in $C_{i}$, so that $r_{i}=\frac{1}{\left|C_{i}\right|}\sum_{j\in C_{i}}d\left(E_{j},r_{i}\right)$
10:  **end for**11:  **until**
$r_{i}$<V (i.e. $r_{i}$ less than the threshold)12:  calculate criterion function $E={\sum_{i=1}^{k}\sum_{p\in C_{i}}\left|p-m_{i}\right|}^{2}$
13: **end for**14: find the minimum of E and get the optimal $k_{opt}$
15: determine the optimal C
16: **return**
C


The main work of our proposed clustering method is to narrow the judgment interval of the optimal *CH* and optimize the selection of the initial convergence center. Therefore, the election of the final *CH* takes into account the energy consumption of ordinary nodes, the energy consumption of the *CH*, and the energy imbalance of boundary nodes. We divide the optimization process of the algorithm into two steps, which are the cluster number selection and the initial cluster center selection.

(1) Determine the number of clusters.

At present, the best number of clusters is selected by iterating the clustering results with different *k* values. Comparing the clustering evaluation function after iteration, the optimal number of clustering is determined [50,51]. The iteration range of the *k* value is the empirical value $2\leq k\leq \sqrt{n}$. However, the number of nodes is too large and conducted over size steps of iterations for *k* value, which seriously affects the real-time performance of the system. According to the real-time requirement of the system, this paper reduces the iteration range of the *k* value and speeds up the selection of the optimal *k*-value. The cluster size $k_{opt}$ is scored by the following formula:

(15) $$k_{\min}=\frac{n_{num}\times (t_{rec}+t_{send}+t_{mcu}+t_{delay})}{T_{C}},$$

(16) $$k_{\min}\leq k_{opt}\leq \sqrt{n},$$

where $T_{C}$ is the time period required by the system, $t_{Send}$ the sending time of the node, $t_{rec}$ is the time when a node receives a message, $t_{\mathrm{mcu}}$ is the time when the master unit processes the data, $n_{num}$ is the number of nodes, $t_{delay}$ is the anti-collision delay, and $k_{\min}$ is the number of clusters that satisfy real-time performance.

The range of cluster number *k* is determined according to the above formula. It not only ensures the real-time nature of the data, but also the rationality of the number of cluster nodes. The optimal *k* value is selected by comparing the clustering evaluation effect function.

(2) Selection of initial convergence centers.

Due to differences in node functions during the monitoring phase, there is a large energy difference between different nodes. This directly affects the choice of the initial cluster center. Therefore, it is necessary to consider the relationship between the initial cluster center and boundary nodes. The initial cluster center is too far away from the boundary node, causing the boundary node to consume more energy for data communication and the node to die prematurely. The initial cluster center is too close to the boundary node to reduce the selectivity of the *CH* rotation and increase the network consumption within the cluster. Therefore, the relationship between the boundary node and the *CH* needs to be considered in the initial cluster center selection.

1. There are *N* boundary nodes in a certain network. The sum of the distances of *l*(l≤N) boundary nodes in this network is less than the optimal communication distance. The optimal cluster center *m* is selected by the farthest boundary node among the *l* boundary nodes. The distance between the reference node and the boundary node, the average energy ratio, and the cosine similarity selected for the optimal clustering center are as follows:

(17) $$S=\frac{a\cdot b}{\left||a||\;||b|\right|},$$

(18) $$P_{i,j,m}(m)=\mu \times \frac{dis_{i,j}}{dis_{i,m}+dis_{j,m}}+\eta \times \frac{E_{m}}{E_{ave}}+\gamma (1-S),$$

(19) μ+η+γ=1,

where a and b are the direction vectors of node *m* relative to the boundary node, S is cosine similarity, $P_{i,j,m}(m)$ is the probability that the node *m* selected as the cluster center, $dis_{i,j}$ is the distance between boundary nodes i and j, $E_{m}$ is the energy of node *m*, $E_{ave}$ is the average energy of the network, and μ,η,γ are the proportion of each part.

2. The distance between the remaining two arbitrary boundary nodes is larger than the optimal communication radius $d_{1hop}$. The boundary nodes farthest from the initial clustering center are selected as the boundary nodes for the next clustering center. The probability of a node being elected as the cluster center is as follows:

(20) $$P(i)=\omega \times \frac{\sum_{m=1}^{N-1}dis_{i,m}}{N-1}+\left(1-\omega \right)\frac{E_{i}}{E_{ave}},$$

where P(i) is the probability that the node i is selected as the cluster center, $dis_{i,m}$ is the distance from boundary node i to initial cluster center m, $E_{i}$ is the energy of node i, $E_{ave}$ is the average energy of network nodes, and ω is the proportion of each part.

3. If *c* ($c<k_{opt}$) cluster centers are selected according to boundary nodes, according to Equation (20), the remaining cluster centers are selected according to any selected cluster centers, and the boundary nodes are replaced by ordinary nodes until the end of $c=k_{opt}$.

The optimal clustering result is obtained from Algorithm 1. However, optimal clustering does not guarantee minimized size. The cluster size is too large to meet the real-time requirements of the system. Therefore, it is necessary to improve the cluster density. If a cluster is relatively dense, the real-time performance of a single *CH* is poor ($n_{i}>n_{\max}$). It is necessary to increase the number of *CH*s to improve the real-time performance of the system. The number of *CH*s is determined as follows:

(21) $$k_{i}=\frac{T_{i}}{\sum_{i=1}^{num}(t_{i\_send}+t_{i\_cpu})+t_{CH}},$$

where *n_i_* is the number of nodes in the cluster, $n_{\max}$ is the maximum number of nodes to satisfy the real-time requirement, $k_{i}$ is the number of clusters, $T_{i}$ is the intra-cluster communication time, $t_{i\_send}$ is the time when a node sends data, $t_{i\_cpu}$ is the central processing unit (CPU) processing time, and $t_{CH}$ is the inter-cluster communication time.

In the above steps, the optimal clustering in the region is obtained. According to the optimal communication distance, the clustering results are hierarchically divided. Data communication between *CH*s is carried out among *CH*s at different levels. When the *CH* energy is below a certain threshold, the *CH* is replaced in the cluster. Nodes close to the optimal *CH* with sufficient energy are selected as the new *CH*s. Depending on different clustering results, the LoRa frequency band in the cluster is replaced and the bandwidth is set. The function of simultaneously performing intra-cluster information communication via different clusters is completed, and the real-time performance of the network is increased. The *CH* is replaced with the same frequency band during inter-cluster communication.

Because of the movement characteristics of the nodes, it is necessary to monitor the status of the current network clustering results in real time. If some nodes in the clustering network have too large a deviation from the *CH*, i.e., $f>f_{\max}$, then the BS re-clusters the node or restructures the whole network. The evaluation function of the restructuring is as follows:

(22) $$f=\underset{i=1,2,\cdots ,k}{\max}\left\{\sum_{\forall n_{i}\in CH_{k}}\frac{d(n_{i},CH_{k})}{N_{num}}\right\},$$

where f is an evaluation factor, $d\left(n_{i},CH_{k}\right)$ is the distance from the node to the corresponding *CH*, and $N_{num}$ is the number of nodes in the cluster.

The BS receives the location and energy information of each node and the clusters through KFNS algorithm, and then sends the clustering information to the nodes. Depending on different clustering results, nodes are divided into *CH*s and ordinary nodes. Ordinary nodes send data information to *CH*s. After data fusion, the *CH* transmits the information to the superior *CH* until the data are transmitted to the BS. The KFNS algorithm adopts a distributed and centralized approach in the cluster routing phase. The BS is not limited by energy and can use high-performance CPUs and large storage devices to perform complex algorithms. 

In the process of node clustering, the BS needs to comprehensively consider the energy imbalance of the boundary nodes, the high energy consumption of *CH* aggregation messages, the compactness of clustering, and the real-time nature of network information. In the case of ensuring the energy consumption of the boundary nodes, the cluster center should be as far as possible from the boundary nodes. In other words, the probability is small that a boundary node is elected as a *CH* when updating a *CH* within a cluster. In order to ensure the compactness of clustering and the real-time nature of network information, it is necessary to limit the number and size of networks. After the clustering is completed, the rotation of the *CH* is completed by the nodes of each cluster. The BS no longer participates in the election of the *CH*, and minimizes the communication energy consumption caused by the *CH* replacement. The BS performs cluster routing operations based on the global information of sensor networks, and evaluates the operation of the whole network according to the real-time data returned by mobile nodes. Once the network clustering compactness is greater than the set threshold, the BS re-clusters the network. The KFNS algorithm can minimize unnecessary network communication and reduce network energy consumption under the condition of ensuring the rationality of clustering.

### 3.3. Recovery Phase

**Figure 5:**
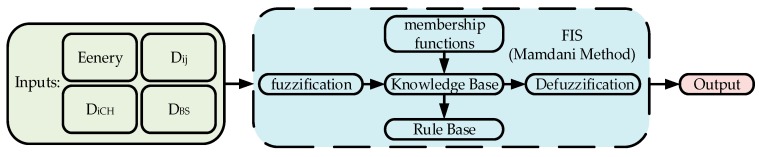
Fuzzy system model.

When the recovery ship moves to a certain range of beacon machine network ($D_{BS}\leq D_{\min}$), the beacon machine needs to be recovered. Considering the recycling efficiency of the nodes, the Dijkstra algorithm is used to plan the recycling path. In order to ensure the real-time performance of the system and reduce the energy consumption of network reorganization, a single-cluster recovery strategy is adopted. After the single cluster node is recovered, the node recovery of the next cluster is performed. The recycling process not only needs to consider the residual energy of the node and the recovery efficiency of the recovery vessel, but also the dynamical change of the *CH* during the recycling process. The order of recycling of nodes is affected by a variety of factors. The best choice of fuzzy rules can be used to obtain better parameter results, as shown in Figure 5. Therefore, this paper optimizes the Dijkstra algorithm by fuzzy logic and adds DFS to optimize the recovery efficiency. The details of the algorithm are shown in Algorithm 2.

**Algorithm 2** Compute node recovery order**Input**: $Energy=\left\{e_{1},e_{2},\cdots ,e_{i}\right\}$, $D_{iCH}=\left\{d_{1CH},d_{2CH},\cdots ,d_{iCH}\right\}$, $D_{ij}=\left\{d_{12},d_{13},\cdots ,d_{ij}\right\}$, $D_{BS}=\left\{d_{1BS},d_{2BS},\cdots ,d_{iBS}\right\}$**Output:** node recycling order1: Min–max normalization technique: $y_{i}=\frac{x_{i}-\underset{1\leq j\leq n}{\min\left\{x_{j}\right\}}}{\underset{1\leq j\leq n}{\max\left\{x_{j}\right\}}-\underset{1\leq j\leq n}{\min\left\{x_{j}\right\}}}$
2: add membership function of fuzzy set3: get inter-node weights and CH chance4: initialize dist[i], visit[θ]
5: **for**
i←n
**do**6:  **if**
!visit[i] && dist[i]<min
7:   min=dist[i]
8:   $\min_{j}=j$
9:  end if10: **end for**11: **for**
j←n do// Relaxed edge12:  **if**
!visit[i] && dist[j] > dist[i] + tab[j][k]
13:   dist[j] = dist[i] + tab[j][k]
14:  **end if**15: **end for**16: get the node to BS minimum weight path17: DFS {18: judging the boundary19: **for**
k←n do20:  DFS (step+1)21: **end for**22: **return}**

In the process of node recovery, a variety of factors work together on the node’s recycling order and *CH* election. To eliminate the dimensional effects between the influencing factors, we use the min–max normalization technique to scale the language variables [52].

(23) $$y_{i}=\frac{x_{i}-\underset{1\leq j\leq n}{\min\left\{x_{j}\right\}}}{\underset{1\leq j\leq n}{\max\left\{x_{j}\right\}}-\underset{1\leq j\leq n}{\min\left\{x_{j}\right\}}},$$

where $y_{i}$ is the normalized value, $x_{i}$ is the given variable, and $\underset{1\leq j\leq n}{\max\left\{x_{j}\right\}}$ and $\underset{1\leq j\leq n}{\min\left\{x_{j}\right\}}$ are the maximum and minimum of all given variables.

**Figure 6:**
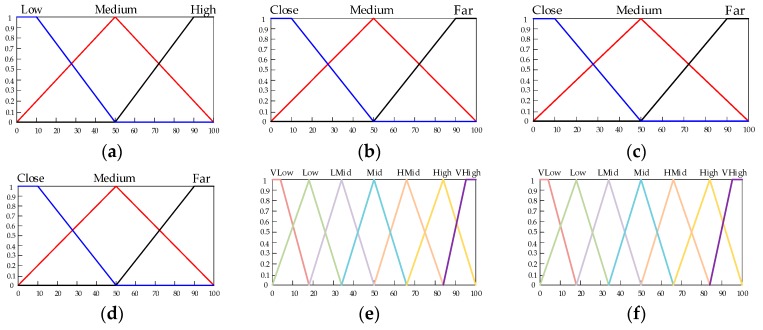
Membership functions of the fuzzy sets. (**a**) Energy; (**b**) $D_{iCH}$; (**c**) $D_{ij}$; (**d**) $D_{BS}$; (**e**) fuzzy output variable weight; (**f**) fuzzy output variable *CH*.

The FLS has four input variables, including node energy (energy), distance between nodes ($D_{ij}$), distance between node and BS ($D_{BS}$), and distance between node and *CH* ($D_{iCH}$). The role of the fuzzifier is mapping each input value to the fuzzy set. Two output variables are generated by the FIS for the chance that the node is elected as the *CH* and for the recycling weight of the node. Weight is related to energy, $D_{ij}$, and $D_{iCH}$, and *CH* election is related to energy, $D_{BS}$, and $D_{iCH}$. The membership function of the input language variable is derived empirically. For each of the four input variables, each input variable has more than one linguistic variable. Figure 6 shows the membership function of each variable. Table 1 and Table 2 are linguistic variables.

The KFNS algorithm uses a fuzzy model to calculate the weights between nodes and elects to collect *CH*s during the recovery phase. The BS uses the weight relationship between nodes to obtain the connected node with the smallest weight of any node. The minimum weight path from any node to the BS is obtained by the Dijkstra algorithm. Similarly, a directed weight map from the BS to the node is obtained using DFS to traverse all nodes to get the optimal path for the reclaimed nodes. In order to ensure the system’s real-time performance and information transmission efficiency, the BS does not communicate directly with nodes in the network; rather, it communicates through the *CH*. The *CH* obtained by the fuzzy model is responsible for collecting the node information of the recovered cluster and forwarding the node information of other clusters.

## 4. Simulation and Experiments

In order to verify the feasibility of the algorithm, the proposed algorithm was simulated and verified by MATLAB on an Intel Core 3.9-GHz CPU with 8 GB memory, with different requirements in each stage of the marine observation beacon recovery process, in terms of computing power between nodes and BS. In the stage of recycling, network operations do not involve the participation of BS. The node uses a low-power CPU design that greatly limits the computing power of the node. Therefore, in the monitoring phase, we compared algorithms that include LEACH-C, DEEC, CECA, and EEUC. In the cluster routing phase, the addition of BS greatly increases the computing power of the network, allowing it to run more complex algorithms. Therefore, the algorithm proposed in the EBRP and Reference [35] is compared in the cluster routing phase. We call the algorithm proposed in Reference [35] the *k*-means *CH*. In the recovery phase, the effects of three optimal recovery strategies, namely, optimal distance, optimal energy, and fuzzy logic, on recovery efficiency and node energy are compared. The simulation parameters are shown in Table 3.

### 4.1. Monitoring Phase Simulation

According to the actual situation, the nodes are distributed across an area of varying size. The node communication range may cover the whole distribution area, or it needs multi-hop routing to complete area coverage. Both cases were simulated and analyzed.

#### 4.1.1. Single-Hop Coverage Simulation

**Figure 7:**
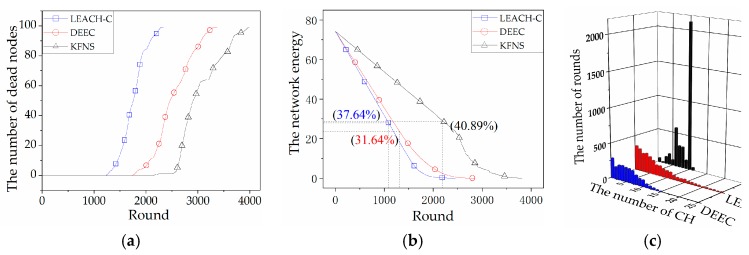
(**a**) The curves of dead node number by rounds; (**b**) network energy consumption; (**c**) number of cluster heads and boundary nodes.

The simulation environment was within the distribution area of nodes, and any two nodes could communicate with each other. That is, all nodes were within the maximum communication range of any node. Figure 7a compares the network life cycles of the three algorithms: LEACH-C, DEEC, and KFNS. The difference between the number of dead nodes and the time between algorithms can be seen. Compared to the former three algorithms, KFNS had a longer network life cycle. The number of rounds of the first dead node of the KFNS algorithm was 2195. The network life cycle increased by 201% and 160% compared to LEACH-C and DEEC. The algorithm proposed in this paper uses a distributed algorithm in the monitoring phase to reduce the energy loss caused by global network communication. The tasks of this phase were completed through the information exchange of some nodes. Figure 7b compares the relationship between the total energy of the network and the time of the three algorithms by comparing the remaining states of the network energy when the first node in the graph dies. The algorithm proposed in this paper has the longest survival time, and the total network has the most residual energy. The excess of network energy compared to other algorithms can be ignored relative to the prolongation of network lifetime. Figure 7c compares the number of *CH*s and boundary nodes generated by each algorithm during operation. The more the algorithm concentrates on the generated data, the more stable the number and size of clusters in the algorithm will be. From the data in the figure, it can be concluded that the mobility of the node seriously affects the LEACH-C and DEEC algorithms. There was a big difference in cluster size throughout the network life cycle. The algorithm proposed in this paper uses local network information for clusters to interact with each other. In this case, we assume that the physical boundary of the network does not change much, and that the number of boundary nodes does not vary greatly.

#### 4.1.2. Multi-Hop Coverage Simulation

**Figure 8:**
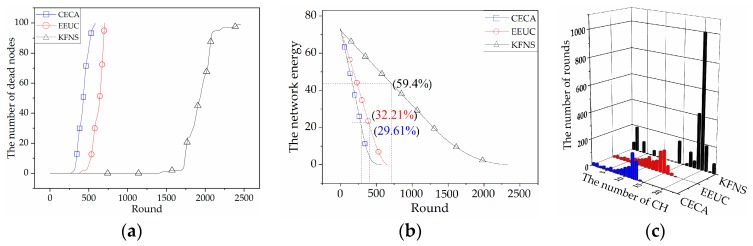
(**a**) The curves of dead node number by rounds; (**b**) network energy consumption; (**c**) number of cluster heads and boundary nodes.

The simulation used a large set of randomly distributed nodes, whereby any two nodes may need other nodes to forward information for communication. Figure 8a compares the network node lifetime of the multi-hop clustering routing algorithms CECA, EEUC, and KFNS. Compared with other algorithms, the network lifetime of the KFNS algorithm increased exponentially. The number of rounds of the first dead node of the KFNS algorithm was 1431. The network life cycle increased by 4.88 and 3.56 times compared to CECA and EEUC. Multi-hop networks require more energy for network communication, which leads to excessive consumption of network energy. Through the comparison of the network life cycle, the effectiveness and feasibility of the KFNS algorithm were verified. Figure 8b compares the total energy consumption of the three algorithms over time. The KFNS algorithm has a longer network life cycle and longer network residual energy. Compared to other algorithms that use most of the network energy for inter-node communication, the KFNS algorithm avoids this part of the energy consumption. The network lifetime of KFNS is 3.5 times that of other algorithms. Figure 8c compares the number of *CH*s and the number of boundary nodes generated by the three algorithms in a multi-hop coverage environment. Similarly, CECA and EEUC are affected by node movement. Compared with the single-hop coverage, as the coverage increased, the number of boundary nodes also increased. The concentrated distribution of the number of boundary nodes indicates that the KFNS algorithm can cope with the impact of node movement, and the network has higher stability.

In this section, the application of the KFNS algorithm in different ranges was simulated by MATLAB. From the simulation results, compared with other algorithms, the KFNS algorithm could greatly improve the lifetime of network nodes and the network energy consumption. It is particularly useful for large-scale multi-hop routing networks; because of the reduction of large-scale communication loss, the algorithm has better performance in saving network energy.

### 4.2. Cluster Routing Phase Simulation

**Figure 9:**
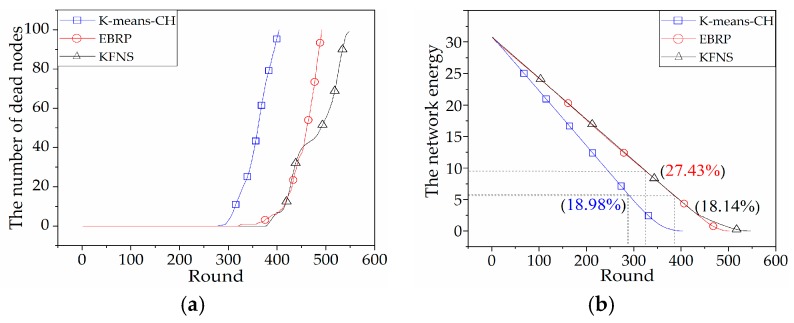
(**a**) The curves of dead node number by rounds; (**b**) network energy consumption.

This phase was a simulation of the node networking and information collection process to compare the differences in network lifetime and network energy consumption of each algorithm. Figure 9a compares the node lifetimes of the *k*-means *CH*, EBRP, and KFNS algorithms. The number of rounds of the first dead node of the KFNS algorithm was 386. The network life cycle increased by 130% and 121% compared to k-means *CH* and EBRP. The k-means *CH* algorithm requires frequent network reorganization and *CH* replacement. The algorithm does not limit the cluster size and affects the real-time performance of the system. The EBRP algorithm performs clustering and then performs *CH* election and communication. The choice of the initial clustering center of the EBRP algorithm is only iteratively selected based on the Euclidean distance. The energy difference between nodes is ignored. The energy-unbalanced node may be close to or far away from the *CH* while ensuring that the *CH* is optimal. The node close to the *CH* causes selective reduction of the *CH* rotation, which increases the consumption of the common node. The node of energy imbalance being far away from the *CH* causes the node energy to be further consumed, and the node dies prematurely. Therefore, the clustering result has contingency, which directly affects the stability of network survival time. Figure 9b compares the total energy consumption of the network using the three algorithms. The KFNS algorithm has a lower residual network energy. The proposed algorithm has a positive effect on solving WSNs with extremely unbalanced node energy. Figure 9b also reflects the excessive network energy surplus of the EBRP algorithm. The algorithm does not consider the relationship between the *CH* and lower-energy nodes. As a result, lower-energy nodes die early, and there remains a large amount of energy in the cluster.

The lifetime and the energy consumption of the KFNS algorithm and other clustering algorithms in the cluster routing phase were also compared by MATLAB simulation. The proposed algorithm has advantages in terms of system real-time performance, energy consumption balance, and node clustering rationality. In the *CH* election stage, the location relationship between the optimal *CH* and the boundary nodes (energy-unbalanced nodes) is considered to make the *CH* election more reasonable.

### 4.3. Recovery Phase Simulation

**Figure 10:**
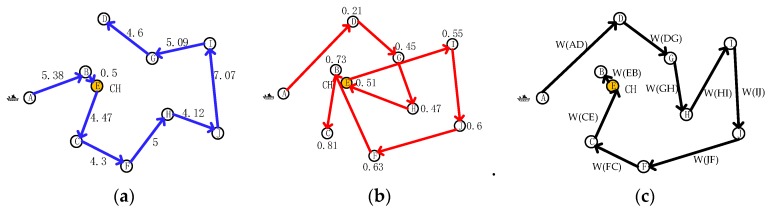
Path comparison of different recycling strategies. (**a**) Optimal path.; (**b**) Optimal energy; (**c**) KFNS algorithm.

**Figure 11:**
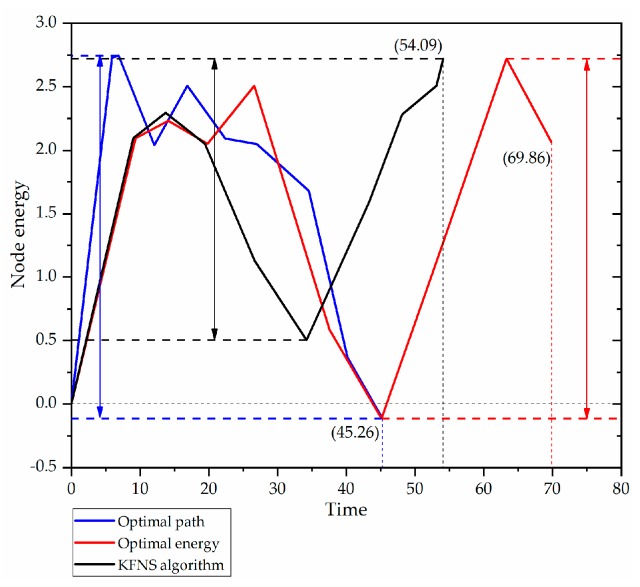
Comparison of recycling efficiency.

The recycling order of nodes directly affects the recycling efficiency. Through the simulation of the recycling process, the rationality of the proposed algorithm in the recycling process was verified. Figure 10 shows the comparisons of optimal path, optimal energy, and the KFNS algorithm. The energy, node position, and *CH* rotation order of each node were the same in the simulation process, and the energy consumption followed Reference [45]. By comparing the two parameters of residual energy and recovery time, the advantages and disadvantages of recovery strategy can be discussed. Figure 11 compares the node recovery time of the three path plans with the remaining energy of the recovered nodes. The path optimization and energy optimization had dead nodes in the recovery process. The optimal path and optimal energy network energy fluctuation range were 2.857 and 2.835, and the KFNS algorithm had an energy fluctuation range of 2.219. Therefore, compared with other path selection methods, the KFNS algorithm performs better in balancing node energy consumption and reducing recovery time.

### 4.4. Node Recycling Process Simulation

**Figure 12:**
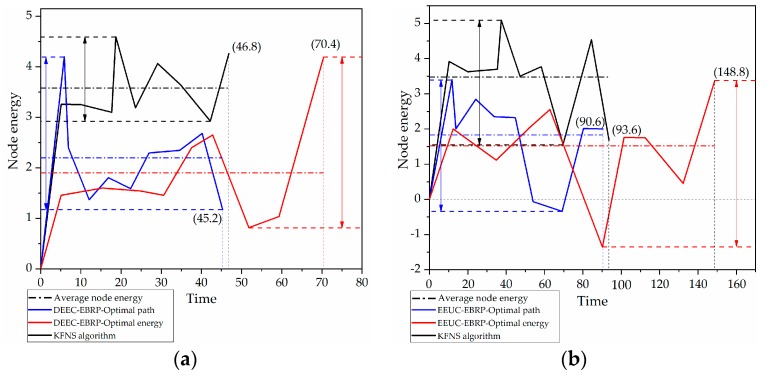
Comparison of node recovery results. (**a**) Single-hop coverage. (**b**) Multi-hop coverage.

Because the recycling process of the marine observation beacon is divided into multiple stages, and the computing power of the network in each stage is quite different, several representative algorithms need to be combined when performing the node recovery process simulation. It can be concluded from Figure 7 that the DEEC algorithm in the single-hop coverage simulation had better performance. In the multi-hop coverage simulation results in Figure 8, the EEUC algorithm performed better. Through the simulation results of the cluster routing stage in Figure 9, it can be concluded that the EBRP algorithm was more reasonable. We compared the proposed algorithms with several popular algorithms. Due to the different deployment scope of the marine observation beacon, we divided the simulation environment into single-hop coverage and multi-hop coverage. Figure 12a shows a comparison of recovery results for single-hop coverage. The average residual energy of the recovered nodes in the proposed solution was 1.6 times and 1.88 times that of other algorithms. Moreover, it had better performance in balancing network energy consumption. Figure 12b shows a comparison of recovery results for multi-hop coverage. The average energy of the KFNS algorithm recovery node was 1.9 times and 2.2 times that of other methods. This also illustrates the potential of the proposed algorithm for the recovery of large-scale nodes. Compared with other algorithms where node death occurs, the nodes recovered by the KFNS algorithm had a certain energy surplus. Based on the simulation results, the KFNS algorithm can provide better performance in response to large-scale node recovery.

### 4.5. Implementation and Experiments

**Figure 13:**
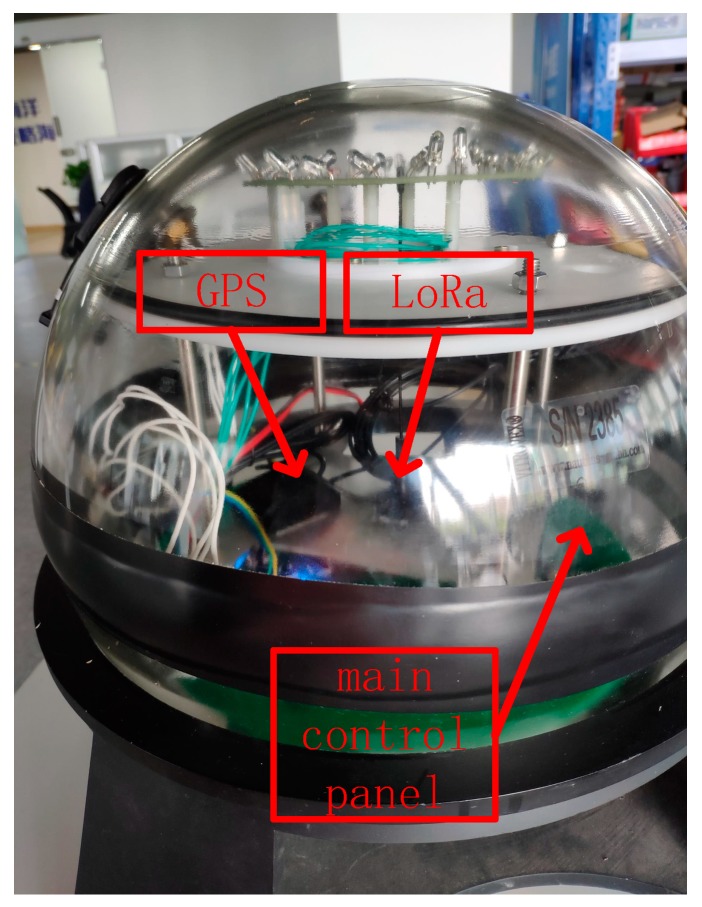
Beacon structure.

**Figure 14:**
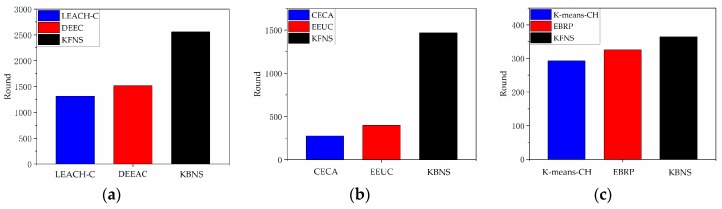
Comparison of network lifetime of each algorithm: (**a**) single-hop network environment; (**b**) multi-hop network environment; (**c**) network routing phase.

We verified the feasibility of the proposed algorithm and other algorithms through the hardware platform shown in Figure 13. The platform performed channel monitoring and data communication through the UM402 LoRa module, and the positioning and calibration time were completed by the NEO-6M-0-001 GPS module. The experimental area was 100 m×100 m×π without a building covering a circular area. The optimal communication distance of the experimental platform was 50 m, and the network time period was 10 s. The node received, sent, and processed data at 0.6 s/round. Equation (11) had an energy ratio of 0.8 and a BCH selection threshold of 0.3, which led to α=0.6,β=0.2,γ=0.2 in Equation (13) of the temporary network routing. In Algorithm 1, Equation (18) for selecting the cluster center led to μ=0.3,η=0.5,γ=0.2, and ω=0.2 in Equation (20); the cluster center iteration threshold was 0.1, and the network reorganization evaluation function threshold was set to 8. The experiment was carried out 15 times, and the average experimental results are shown in Figure 14. The experimental results show that the KFNS algorithm provides better performance than the other algorithms because it solves practical problems in stages.

## 5. Conclusions

A large amount of equipment is required for environmental monitoring during the marine development process. However, in the process of recycling marine monitoring equipment, due to the influence of natural factors such as currents and tides, the network is unevenly distributed, leading to communication delay and packet loss. Due to the complexity of the marine environment, the energy storage of monitoring equipment is limited and cannot be supplemented. This brings difficulties and challenges to the recycling of marine equipment. 

In this paper, a clustering algorithm for WSNs based on the *k*-means algorithm and FLS was proposed for the recovery of marine observation beacons. The KFNS algorithm was divided into three phases: monitoring, cluster routing, and recovery. In the monitoring phase, network consumption was reduced, and network energy was saved to the maximum extent. In the cluster routing phase, according to the real-time requirements of the system and the location information of the boundary nodes, the clustering size and the initial clustering center were determined, which reduced the number of cluster iterations and made the clustering more practical. In the process of *CH* selection, the optimal cluster center location, and the energy difference between common nodes and boundary nodes were considered comprehensively. In the recovery phase, a fuzzy model was used to calculate the weight between nodes, and the *CH* was selected. The Dijkstra algorithm and DFS were used to determine the optimal recovery path of the nodes. The proposed KFNS algorithm has three main advantages over other methods. Firstly, centralized and distributed algorithms improve the design procedures and increase the practicality of the algorithm’s practical application. Secondly, the algorithm proposed in this paper has a lower requirement for node hardware and it reduces production costs. Thirdly, fuzzy rules can break through the limitations of traditional assessment methods. The simulation and experimental results showed that the proposed KFNS algorithm has lower network consumption, a longer network lifetime, and a more efficient recovery strategy.

For marine monitoring equipment recycling scenarios, collaboration between multiple BSs leads to information intersections and repetitions. Therefore, in the future, we will further study the information sharing of multi-base station cooperation. The path planning problem of multiple BSs is also one of the future research directions.

## Figures and Tables

**Table 1:** Linguistic variables.

Energy	$D_{ij}$	$D_{BS}$	$D_{iCH}$
Low	Close	Close	Close
Medium	Medium	Medium	Medium
High	Far	Far	Far

**Table 2:** Linguistic variables.

No.	Input				Output	
	Energy	$D_{iCH}$	$D_{ij}$	$D_{BS}$	Weight	*CH*
1	Low	Close	Close	Close	Low	HMid
2	Low	Close	Medium	Medium	HMid	Mid
3	Low	Close	Far	Far	High	LMid
4	Low	Medium	Close	Close	Low	LMid
5	Low	Medium	Medium	Medium	LMid	Low
6	Low	Medium	Far	Far	Mid	VLow
7	Low	Far	Close	Close	VLow	LMid
8	Low	Far	Medium	Medium	VLow	Low
9	Low	Far	Far	Far	Low	VLow
10	Medium	Close	Close	Close	HMid	High
11	Medium	Close	Medium	Medium	High	HMid
12	Medium	Close	Far	Far	VHigh	Mid
13	Medium	Medium	Close	Close	HMid	Mid
14	Medium	Medium	Medium	Medium	High	LMid
15	Medium	Medium	Far	Far	VHigh	Low
16	Medium	Far	Close	Close	VLow	LMid
17	Medium	Far	Medium	Medium	Low	Low
18	Medium	Far	Far	Far	LMid	VLow
19	High	Close	Close	Close	Mid	VHigh
20	High	Close	Medium	Medium	HMid	High
21	High	Close	Far	Far	VHigh	HMid
22	High	Medium	Close	Close	Mid	HMid
23	High	Medium	Medium	Medium	High	Mid
24	High	Medium	Far	Far	VHigh	LMid
25	High	Far	Close	Close	Low	High
26	High	Far	Medium	Medium	LMid	Mid
27	High	Far	Far	Far	HMid	LMid

**Table 3:** Simulation parameters.

Parameter Name	Parameter Value
Single-hop network size $\left(R_{s}\right)$	30 m×30 m×π
Multi-hop network size $\left(R_{m}\right)$	100 m×100 m×π
Number of nodes (n)	100
Initial energy $\left(E_{0}\right)$	$E_{i}=E_{0}\times (0.9+rand*(0.1))$, $E_{0}=5$
Communication range of sensors (r)	60 m
Time for each round (T)	10 s
Speed range ($V_{l}$)	1–5 m/s
Energy consumption of transmission circuit $\left(E_{elec}\right)$	50 nj/bit
Amplifier parameter for free-space model $\left(E_{\varepsilon fs}\right)$	10 $pj/bit/m^{2}$
Amplifier parameter for multi-path model (εmp)	0.0013 $pj/bit/m^{4}$
